# In Vivo Irradiation of Mice Induces Activation of Dendritic Cells

**DOI:** 10.3390/ijms19082391

**Published:** 2018-08-14

**Authors:** Eszter Persa, Tünde Szatmári, Géza Sáfrány, Katalin Lumniczky

**Affiliations:** Department of Radiation Medicine, Division of Radiobiology and Radiohygiene, National Public Health Institute, Anna u. 5, 1221 Budapest, Hungary; persa.eszter@osski.hu (E.P.); szatmari.tunde@osski.hu (T.S.); safrany.geza@osski.hu (G.S.)

**Keywords:** dendritic cells, ionizing radiation, cytokine secretion, T cell activation, antigen uptake, antigen presentation

## Abstract

It is becoming clear that ionizing radiation positively influences certain immune parameters, which opens the possibility for combining radio- and immunotherapies in cancer treatment. The presence of functionally competent dendritic cells (DCs) is crucial in mounting a successful antitumor immune response. While it has been shown that DCs are relatively radioresistant, few and contradictory data are available on how ionizing radiation alters the functional integrity of these cells. Therefore, our objective was to investigate the effect of whole-body irradiation on the function of splenic DCs. C57Bl/6 mice were irradiated with 0.1, 0.25, and 2 Gy X-rays and changes in the phenotype of splenic DCs were compared to unirradiated controls. An increase was seen in DC surface markers influencing DC-T cell interactions. In vivo cytokine production was determined by direct intracellular cytokine staining. Irradiation with 2 Gy induced a 1.6-fold increase in IL-1α production, while the combination of irradiation and lipopolysaccharide (LPS) treatment induced a 3.9-fold increase, indicating a strong synergism between irradiation and LPS stimulation. Interaction of DCs with effector and regulatory T cells was investigated in a mixed lymphocyte reaction. While DCs from control animals induced stronger proliferation of regulatory T cells, DCs from animals irradiated with 2 Gy induced stronger proliferation of effector T cells. Antigen uptake and presentation was investigated by measuring the capacity of DCs to internalize and present ovalbumine (OVA)-derived peptides on their major histocompatibility complex (MHCI) molecules. Irradiation with 2 Gy did not influence antigen uptake or presentation, while low doses stimulated antigen uptake and reduced the level of antigen presentation. In conclusion, high-dose in vivo irradiation induced increased expression of T cell costimulatory markers, enhanced production of proinflammatory cytokines and a stronger stimulation of effector T cell proliferation than that of regulatory T cells. However, it did not influence DC antigen uptake or presentation. On the other hand, low-dose irradiation increased antigen uptake and lowered antigen presentation of DCs, indicating that low- and high-dose irradiation act on different pathways in DCs.

## 1. Introduction

Radiation was considered as a net immune-suppressing agent for decades [1]. While the exquisite radiosensitivity of the lymphoid system in terms of radiation-induced cell killing is out of the question, recent advances in radiobiology and immunology have made this picture more complex. It was shown that a great variability exists in the radiosensitivity of the different lymphocyte subpopulations [2]. Tumor cells release so-called danger signals in response to radiation, which, in turn, can lead to immune activation and induction of immunogenic cell death [3,4,5]. Irradiation can enhance the production of immune-stimulatory cytokines, and improve infiltration of tumors with lymphocytes. Most importantly, these immune-modulatory effects are not only restricted locally to the tumor site but manifest at a systemic level [6]. Several clinical observations have been published supporting the immune-stimulatory effect of radiation [7,8]. These findings opened the gate for studies exploring optimal combinations of radiotherapy and immunotherapy in order to achieve a synergistic effect.

Dendritic cells (DCs) are indispensable for mounting a successful immune response and have been extensively studied in various immune-therapeutic trials. A DC-based vaccine was shown to be effective in a phase III clinical study for the treatment of advanced prostate cancer, and was approved for use in clinic [9]. The synergistic effects of radiation therapy and DC immunotherapy have been documented in patients with oesophageal cancer [10] and prostate cancer [11]. However, in order to further improve the efficiency of these combined therapies, we need to know how irradiation influences the functional parameters of dendritic cells. 

The increasing exposure of the population to low-dose ionizing radiation (mainly in the medical sector or through various environmental sources) is becoming a major epidemiological concern worldwide mainly due to the possible role of low-dose irradiation to enhance risk of radiation-induced carcinogenesis [12]. Low doses are considered doses below 0.1 Gy [13]. Low-dose irradiation has immunomodulatory effects by affecting the phenotype and function of several immune cells, such as macrophages, various T cell subsets, and DCs [14,15]. Low-dose-induced immune modulation influences antitumor-immune surveillance and thus affects the risk of radiation-induced carcinogenesis as well. Despite the fact that research directed towards low-dose-radiation effects on the immune system has substantially increased during the last few years [14,16,17,18,19,20,21], still very little is known about the main immune cell targets of radiation, the dose-response of changes, or the impact of radiation on the functional parameters of immune cells. 

We have previously studied the effect of in vivo irradiation on splenocyte subpopulations and showed that different cell populations had different sensitivities to low doses [2]. DCs, along with regulatory T cells (Tregs), were among the most radioresistant splenocyte subpopulations, with low-dose radiation inducing a much lower rate of apoptosis than in other splenocyte subpopulations. In another study, we investigated radiation effects on the functional parameters of Tregs and proved that although the Treg pool was much less affected after irradiation compared to other splenocyte subpopulations, the functional integrity of Tregs was still compromised after irradiation with 2 Gy [22]. In the present paper, the objective of the study was to examine how irradiation affected the functional integrity of splenic DCs focusing on changes in cell-surface costimulatory molecules, ability of antigen uptake and presentation, cytokine secretion, and DC-CD4 cell interactions. 

In this paper, we measured changes in DC-specific cell-surface markers, including costimulatory molecules (CD40, CD80, CD86) [23,24], a coinhibitory molecule (B7-H1) [25], and a cell-surface marker involved in antigen capturing and presentation (DEC205) [26]. Cytokines reported to be produced by DCs were selected according to their function in immune regulation and their involvement in acute inflammatory responses. Interleukin-1-alpha (IL-1α) is expressed upon antigen uptake in DCs [27] and together with interleukin-1-beta (IL-1β) plays role in irradiation-induced inflammatory responses [28]. Stimulation of DCs by lipopolysaccharide (LPS) by binding to toll-like receptor 4 (TLR4) and other pathogen-associated molecular patterns (PAMPs) induces strong inflammatory responses and increased IL-1β secretion [29]. Interleukin-6 (IL-6), which is an important mediator of acute-phase response is secreted by DCs and can induce helper T cell generation [30] and CD8+ cell survival [31]. A dose-dependent change was observed in its level after total-body irradiation [32]. Interleukin-10 (IL-10) is an anti-inflammatory cytokine released by DCs upon stimulation of the TLR signalling pathway and supresses CD4+ T cell activation [33]. Tumor necrosis factor-alpha (TNFα) is another cytokine involved in acute-phase response released by DCs upon LPS stimulation [34], which can cause an IL-10-dependent inhibition of CD4+ T cells [35]. Interleukin-12 (IL-12), another cytokine produced by DCs, is involved in Th1 cell differentiation [36] and its level is changing in parallel with DC maturation [37]. 

## 2. Results

### 2.1. High-Dose Irradiation Increased the Expression of T Cell Interaction-Related Phenotypical Markers on Splenic DCs

**Figure 1:**
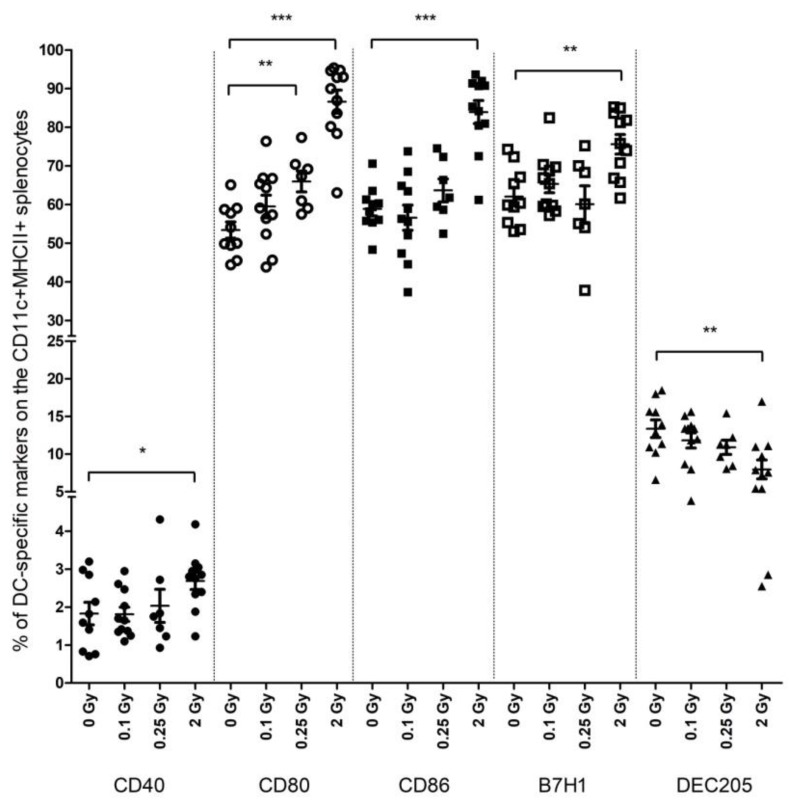
Phenotypical changes on splenic dendritic cells (DCs) after irradiation. Splenocytes isolated 1 day after total-body irradiation of mice were stained with fluorescently labeled antibodies and analyzed by flow cytometry. The figure shows the fraction of DC populations expressing the indicated markers within the total CD11c+MHCII+ splenic DCs. Filled circles represent CD40 samples, empty circles represent CD80 samples, filled squares are CD86 samples, empty squares are B7-H1 samples and triangles represent DEC205 samples. Data represent 10 (0 Gy), 11 (0.1 Gy), 7 (0.25 Gy), and 11 (2 Gy) independent animals. Bars represent mean ± SD. Significance was tested by Student’s *t*-test (* *p* < 0.05, ** *p* < 0.001, *** *p* < 0.0001).

Mice were total-body irradiated with either low- (0.1 and 0.25 Gy) or high (2 Gy)-dose X-rays and, 24 h after irradiation, the phenotype of splenic DCs was investigated by flow cytometry. After irradiation with 2 Gy, the fraction of splenic DCs expressing the CD40, CD80, and CD86 costimulatory, as well as B7-H1 coinhibitory molecules, increased 1.47-, 1.62-, 1.42-, and 1.22-fold, respectively. On the other hand, DEC205-expressing DCs showed a 1.67-fold decrease. While 0.1 Gy did not influence DC phenotype compared to control, sham-irradiated animals, 0.25 Gy induced similar, albeit milder changes in the level of CD80-expressing DCs to 2 Gy (Figure 1). 

In order to determine the durability of this effect, the same phenotypical markers were investigated on DCs 3 days after irradiation but focusing only on 2 Gy irradiation. At this time point, the fraction of CD80-, B7-H1-, and DEC205-expressing DCs tended to normalize, and changes were not significantly different from control animals. However, the fraction of CD40-expressing DCs increased significantly and the higher level of CD86-expressing DCs also persisted (Table 1). 

Thus, it seems that irradiation-induced DC activation was not a transient phenomenon but persisted in animals at least up to 3 days after irradiation. If control DCs were stimulated with LPS, a strong increase in CD40-, CD80-, and CD86-expressing DCs was seen. Similar changes were noted if irradiated DCs were stimulated with LPS, indicating that DC activation upon LPS stimulation was not compromised in the irradiated animals (Table 1).

### 2.2. Low-Dose Irradiation Had Significant Impact on Both Antigen Uptake and Presentation by Splenic DCs

**Figure 2:**
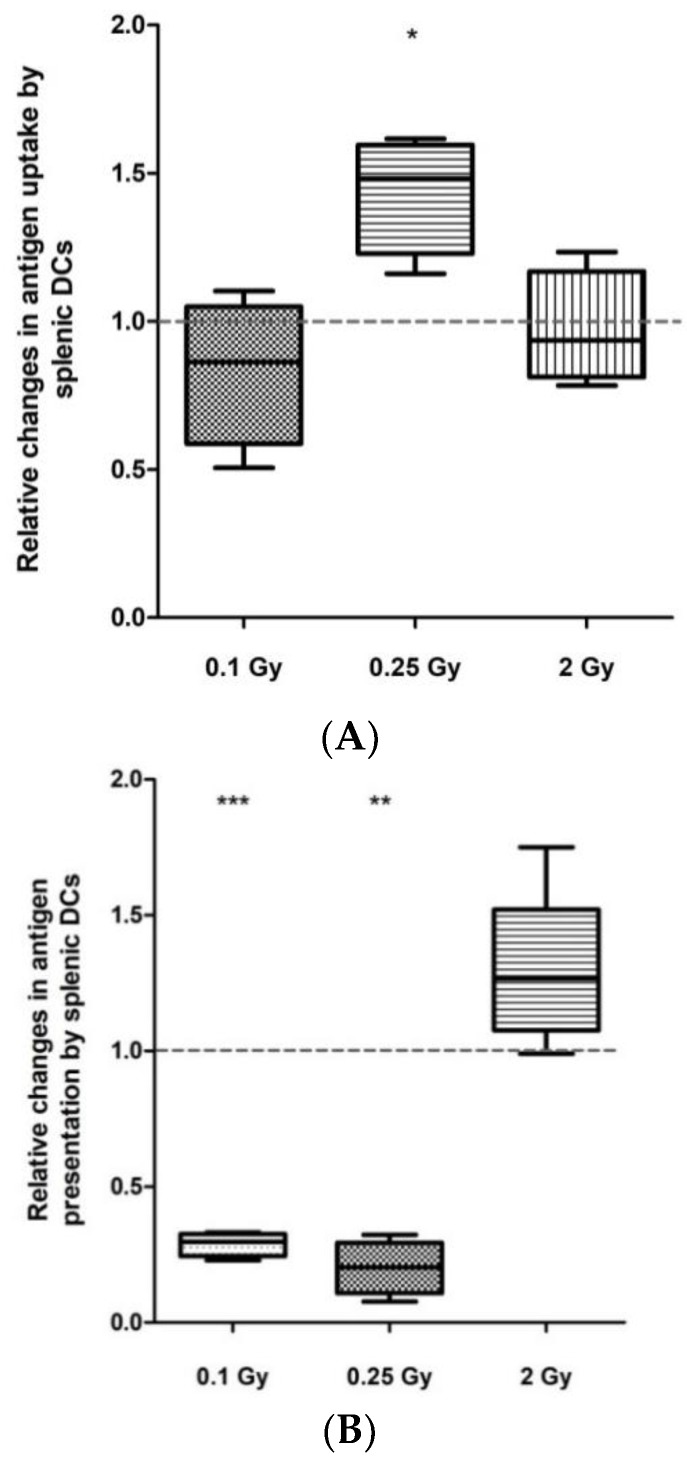
Changes in antigen uptake and presentation by splenic DCs after irradiation. Splenic DCs from total-body irradiated mice were isolated by magnetic cell sorting, as described in Materials and Methods. (**A**) To determine antigen uptake, isolated cells were in vitro incubated with FITC-labeled ovalbumine (OVA) peptide and the fraction of fluorescent DCs was quantified by flow cytometry; (**B**) to determine antigen presentation, isolated DCs were incubated in vitro with the OVA peptide SIINFEKL, stained with fluorescently labeled antibodies directed against OVA peptide-bound MHCI receptors and the fraction of fluorescent DCs was quantified by flow cytometry. Data represent the average of 4 independent experiments, with bars indicating mean ± SD. Significance was tested by Student’s *t*-test (* *p* < 0.05, ** *p* < 0.01, *** *p* < 0.001).

For ex vivo antigen-uptake investigation, purified splenic DCs isolated from mice irradiated 24 h earlier were incubated with a FITC-labeled short peptide of the ovalbumine (OVA) protein. While neither 0.1 Gy nor 2 Gy had any effect, an almost 1.5-fold increase in OVA-peptide uptake was measured in DCs isolated from mice irradiated with 0.25 Gy (Figure 2A and Supplementary Figure S1). 

To investigate the level of antigen presentation, DCs were incubated with unlabeled OVA peptide and stained thereafter with fluorescently labeled antibodies directed specifically against OVA peptide-bound major histocompatibility complex (MHCI) molecules. While, similarly to antigen uptake, 2 Gy did not influence antigen presentation, both 0.1 Gy and 0.25 Gy strongly reduced antigen presentation, leading to a 5-fold decrease in OVA-MHCI level (Figure 2B and Supplementary Figure S2).

### 2.3. Irradiation Increased Cytokine Gene Expression in Splenic DCs

**Figure 3:**
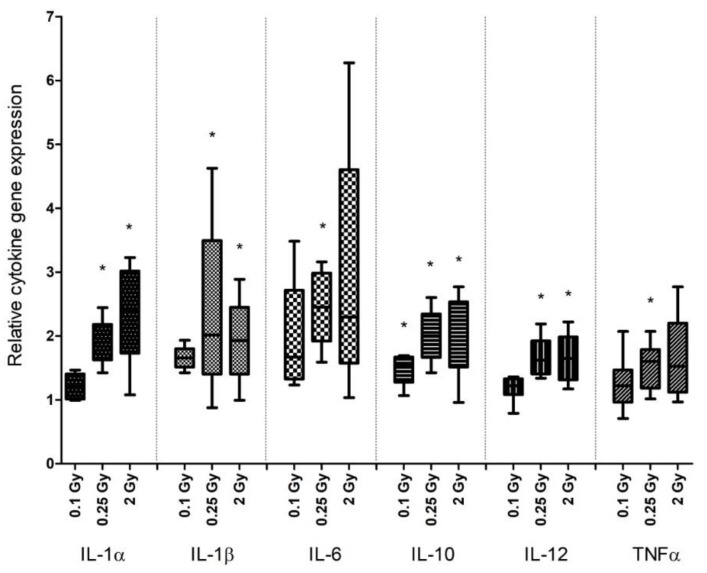
Alterations of cytokine gene expression patterns in splenic DCs after irradiation. Splenic DCs from total-body irradiated mice were separated by magnetic cell sorting, RNA was isolated and the relative expression of the specified cytokines was quantified by quantitative RT-PCR (qRT-PCR) as described in Materials and Methods. The figure shows relative changes in gene expression. Data represent the average of 5 independent experiments, with bars indicating mean ± SD. Significance was tested by Student’s *t*-test (* *p* < 0.05).

Cytokine gene expression was investigated in purified splenic DCs by quantitative RT-PCR (qRT-PCR) 1 day after irradiation. We focused on a panel of cytokines reported to be expressed by DCs (IL-1β, IL-6, IL-10, IL-12, TNFα) [38,39] or known to be influenced by radiation (IL-1α) [40]. Radiation induced upregulation of all of the studied cytokine genes, but the effects were low to moderate, not exceeding a 3-fold increase. The highest level of changes was observed in IL-6 expression, and the least affected genes were IL-12 and TNFα. The expression of IL-1β, IL-6, and IL-10 mildly increased after irradiation with 0.1 Gy, while 0.25 and 2 Gy induced upregulation of all studied cytokine genes. Although gene-expression changes were dose dependent, they did not correlate with the magnitude of the dose, with the exception of IL-1α. TNFα, IL-10, and IL-12 gene-expression levels were very similar after 0.25 Gy and 2 Gy irradiations (Figure 3). 

### 2.4. Irradiation Increased the Production of IL-1α and IL-1β by DCs In Vivo

While changes in cytokine gene-expression levels can be regarded as indicators of radiation exposure, they only reflect functional damage if cytokine protein secretion is also altered. In order to study radiation effects on cytokine production at the protein level in vivo, mice were total-body irradiated, followed by systemic LPS stimulation. Brefeldin A treatment was applied to block transport of cytokines extracellularly. Cytokine production was quantified by direct intracellular cytokine staining in the CD11cMHCII double-positive splenic DCs. This experimental approach was adopted to study the effect of irradiation on DCs in their natural environment. 

**Figure 4:**
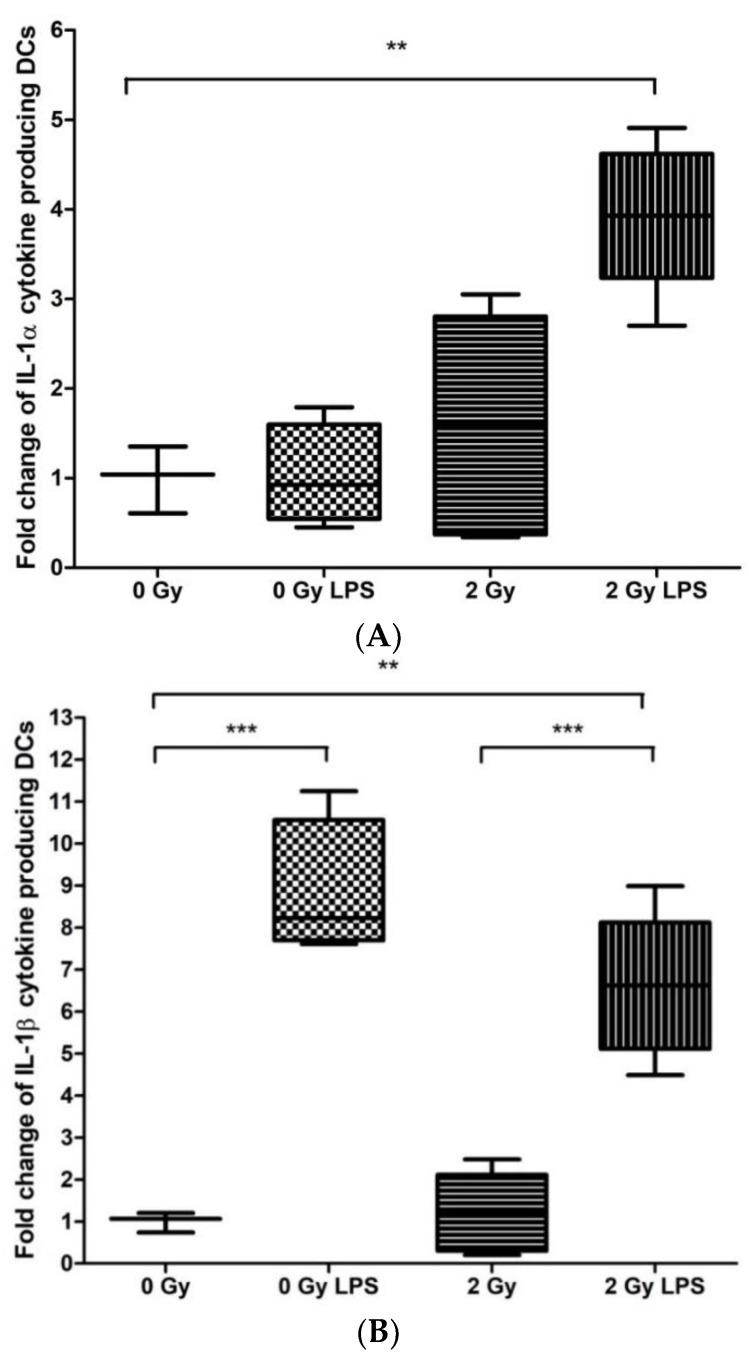
IL-1 production by splenic DCs. Mice were total-body irradiated, treated with an intraperitoneal (i.p.) LPS injection 18 h later, followed by an i.p. Brefeldin A injection. Cytokine production was determined 6 h after LPS treatment in the CD11c+MHCII+ splenic DCs by intracellular cytokine labelling as described in Materials and Methods. The figure shows relative changes in intracellular cytokine levels of (**A**) IL-1α and (**B**) IL-1β. Data represent the average of 5 independent experiments, with bars indicating mean ± SD. Significance was tested by Student’s *t*-test ** *p* < 0.001, *** *p* < 0.0001).

The only cytokines affected by radiation were IL-1α and IL-1β. The fraction of IL-1α producing splenic DCs in sham-irradiated control mice was low, varying between 0.6% and 1.5% of CD11c+MHCII+ cells and LPS treatment did not change the level of IL-1α production. Irradiation of mice with 2 Gy induced a 1.75-fold increase in DCs producing IL-1α but, due to the high variation between animals, results were statistically not significant. LPS treatment of mice irradiated with 2 Gy induced a strong increase (4.3-fold compared to sham-irradiated and 2.5-fold compared to mice irradiated only) in IL-1α production (Figure 4A and Supplementary Figure S3A).

IL-1β levels, similarly to IL-1α levels, were very low in control, sham-irradiated animals, varying between 0.59% and 0.96% of splenic DCs. However, in contrast to IL-1α, LPS treatment lead to a strong, almost 9-fold increase in IL-1β, reflecting the response of splenic DCs to the acute systemic stress induced by LPS. Irradiation itself did not change IL-1β levels compared to control animals. However, if irradiated mice were treated with LPS, IL-1β production increased 6.6-fold and this value was not statistically different from that found in the DCs of control mice treated with LPS (Figure 4B and Supplementary Figure S3B). 

### 2.5. Irradiated DCs Stimulated Teff and Inhibited Treg Cell Proliferation

**Figure 5:**
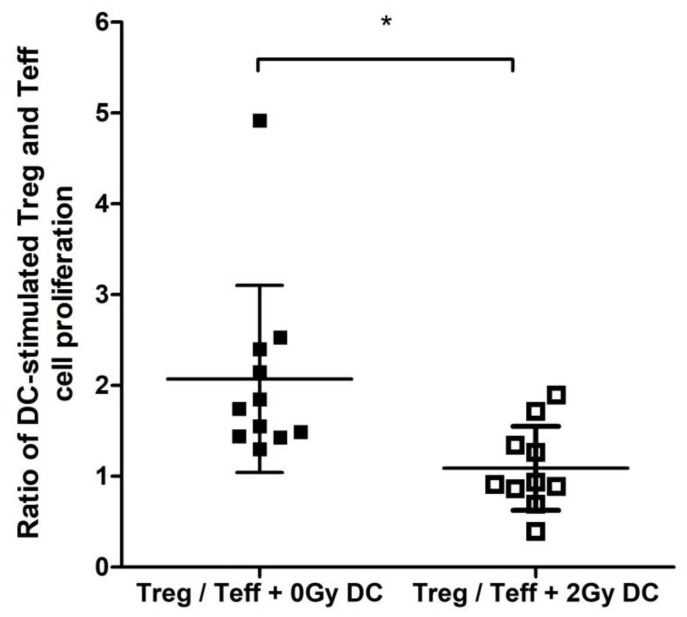
DC-induced proliferation of CD4 effector and regulatory T cells. Splenic DCs from irradiated and control mice as well as CD4+C25− effector and CD4+CD25+ regulatory T cells from nonirradiated mice were isolated by magnetic cell sorting. DCs were in vitro cocultured with either effector T cells or regulatory T cells and T cell proliferation was evaluated by ^3^H-thymidine incorporation. The figure shows the ratio of cell proliferation between regulatory and effector T cells cocultured with DCs from either control or irradiated mice. Data represent the average of 3 independent experiments with DCs isolated from 2–4 independent mice/experiment, with bars indicating mean ± SD. Significance was tested by Student’s *t*-test with * *p* < 0.005.

Splenic DCs isolated from sham-irradiated mice or mice irradiated with 2 Gy were cocultured in vitro with either purified CD4+CD25− effector T cells (Teffs) or purified CD4+CD25+ Tregs and cell-proliferation rate was measured 5 days later (Supplementary Table S1). The proliferation rate of Tregs incubated with DCs from control mice was 2.1-fold higher than the proliferation rate of Teffs incubated with the same DC population. If Tregs and Teffs were incubated with DCs from mice irradiated with 2 Gy, no difference was seen in the proliferation rate of the two cell populations (Figure 5).

In order to see whether irradiation specifically stimulated Teff proliferation or suppressed Treg proliferation, we calculated the ratio between proliferation rate of T cells in the presence of DCs from irradiated and control mice. This fraction was 0.68 for Treg + 2 Gy DCs/Treg + 0 Gy DCs and 1.39 for Teff + 2 Gy DCs/Teff + 0 Gy DCs, indicating that irradiated DCs preferentially shifted the balance of CD4 proliferation in favour of Teff proliferation (Supplementary Figure S4). 

## 3. Discussion

Functionally competent DCs play a pivotal role in the antitumor immune response. This is the reason why several of the immunotherapeutic approaches already applied in clinics or in various phases of clinical trials include DC-based approaches. Recently it has been reported that radiotherapy can positively influence the antitumor immune response and thus could act in synergy with certain immunotherapeutic modalities [41,42,43]. There are data indicating that the potential of irradiation to augment antitumor immunity depends on DC activation [44]. Low-dose radiation exposure constitutes another important factor with potential immune modulatory effects. While the direct cytotoxic effect of low doses is of less concern, they can substantially influence the functional performance of the immune system. However, the mechanisms of how irradiation influences DC function, or the dose dependency of these effects, are not yet clarified, and available data are often contradictory.

It was shown by several groups, including ours, that DCs were among the most radioresistant cells within the peripheral blood and they were much less prone to radiation-induced apoptosis than other peripheral blood leukocytes [2,45,46,47]. Formerly, we demonstrated an approximately 30% reduction in the pool of splenic DCs after irradiation with low doses (0.01 Gy and 0.1 Gy) and showed that the effect was relatively persistent. However, since DC apoptosis was not increased after these doses, changes in the splenic DC pool were most probably the result of a redistribution of the DC population rather than a direct cytotoxic effect [2]. 

In the present study, we focused on investigating the effect of ionizing radiation on the functional integrity of splenic DCs identified as MHCII^high^CD11c cells. Our aim was to study the effect of irradiation on DCs in their natural environment; thus, mice were subjected to total-body irradiation and DCs were analyzed immediately upon isolation from the animals. Radiation doses were selected based on their relevance in various medical applications. Two Gy is the conventional dose/fraction used in cancer radiotherapy and it is the most often used as a reference dose in various radiobiological studies. Doses between 0.25 and 1 Gy are routinely used by several medical centres for the treatment of various chronic inflammatory conditions of the joints [48]. Doses up to 0.1 Gy can be acquired during medical-diagnostic procedures employing radiation exposure (i.e., whole-body CT scans, positron-emission tomography, or interventional radiology). Although local- or partial-body irradiation is generally used in clinical situations, it has been shown by Ventura et al. that even local irradiation can elicit systemic immune responses and can modulate DC function at systemic level [49]. 

Here we report a radiation-induced activation of splenic DCs evidenced by an increase in the fraction of DCs expressing phenotypical markers responsible for an efficient DC–T cell interaction (CD40, CD80, CD86) and by an increase in the expression of several cytokines. Importantly, while this effect was clearly present after high-dose (2 Gy) irradiation, low doses also had certain stimulatory effects on DC activation (higher fraction of CD80-expressing DCs, as well as increased cytokine expression, either after 0.1 Gy, after 0.25 Gy, or both). 

We also showed that irradiation did not compromise the capacity of DCs to react to LPS stimulation since production of IL-1α and IL-1β was maintained or increased after irradiation even though the fraction of DCs producing the respective cytokines was low. We think the main reason for the low detected level of cytokine-producing DCs in vivo resides in our experimental conditions. Traditionally intracellular cytokine measurements were optimized for in vitro cultures. Adopting these experimental conditions to in vivo settings has been reported in several scientific publications [50,51] but it is not very frequently used because it is difficult to optimize the concentration of LPS and Brefeldin A required for DC stimulation and the blocking of protein transport in vivo. Thus, we think the low levels of cytokine-producing DCs are due to the fact that the efficiency of in vivo LPS stimulation and Brefeldin A blocking is inferior to that achievable under in vitro conditions. 

Available literature is relatively contradictory on whether ionizing radiation induces or inhibits DC activation. Our data are consistent with those published by Gupta et al., who showed increased activation (elevated levels of CD86 and CD70 expression) of tumor-infiltrating MHCII^high^CD11c murine splenic DCs after high (10 Gy), single-dose local tumor irradiation [44]. Most publications, though, investigated in vitro matured bone-marrow-derived murine or monocyte-derived human DCs, where irradiation was also performed in vitro. Increased CD40 and CD86 expression was reported on DCs derived from monocytes irradiated with 5 Gy, but irradiation did not influence DC cytokine production [52]. Liao et al. reported no changes in the expression of costimulatory molecules of bone-marrow-derived murine DCs after high-dose irradiation [53]. Several groups reported reduced DC activation after 20 Gy or higher single-dose irradiation, which we think is due to the very high doses used [46,54,55]. Jahns et al. though, using very similar doses to ours, albeit delivered in a fractionated schedule, reported basically no changes in the cell-surface activation markers or cytokine secretion capacity of immature or LPS-matured monocyte-derived human DCs [56]. On the other hand, Malecka et al. found comparable levels to our data of cytokine secretion in human DCs after irradiation [47]. In our opinion, the main reason for the high variability in the data consists in the big differences in DC populations and experimental conditions used. Another reason for this variability might rely on the fact that most studies were carried out under in vitro conditions, DC monocultures being irradiated, and their response to ionizing radiation investigated. However, under in vivo conditions, where both DCs and their microenvironment are irradiated, DC responses to irradiation might be different. This is nicely demonstrated by Malecka et al., who showed that, in a DC-fibroblast coculture, irradiating DCs only led to a significant reduction in the Th17 activating capacity of DCs. However, if fibroblasts were also irradiated, they could restore DC functions [47]. 

DC activation induced by 2 Gy had a significant impact on DC–CD4 cell interaction as well. While Treg proliferation was stronger than Teff proliferation after incubation with DCs from nonirradiated mice, this was reversed if DCs from mice irradiated with 2 Gy were used. This means that irradiation stimulated DC–Teff rather than DC–Treg interactions, potentially contributing to the development of an immune activation. Similarly to the high variability in the data regarding ionizing radiation’s effects on DC phenotypes, no consensus exists on how ionizing radiation influences DC–T cell interactions either. Depending on the experimental conditions, type, and source of investigated DCs and irradiation doses used, some papers report that irradiated DCs stimulate T cell proliferation [55,57], while others report that T cell proliferation is inhibited [52,54,56,58,59]. We have not found any data, though, which specifically compared the effect of irradiated DCs on Teff and Treg cells. 

Another important functional characteristic of DCs is their antigen uptake and presentation capacity. This was studied by measuring either the uptake of a short, OVA-derived peptide or its MHCI-derived presentation on DCs. We showed that, while high-dose irradiation did not influence any of these two parameters, low doses did. It cannot be excluded that variations in DC-antigen presentation were due to radiation-induced changes on MHCI molecule levels on DCs. It has been reported that coculture of DCs with irradiated tumor cells or tumor antigens increase DC antigen uptake and presentation [60]. Similarly, irradiation induced increased MHCI levels on various tumor cells [61,62]. However, very few publications studied the direct effect of irradiation on DC antigen uptake or presentation. Liao et al. demonstrated that high-dose irradiation specifically improved presentation of an MHC class I-restricted exogenous short peptide by DCs, and this process was not due to ionizing radiation-induced alterations on DC MHCI levels [53]. Data on low-dose ionizing-radiation effects on these functional characteristics of DCs are missing, though. By increasing antigen uptake and decreasing antigen presentation, low doses seem to induce a shift in the splenic DC population towards a less mature state. The reason why high-dose irradiation does not, while low-dose irradiation does damage DC antigen uptake and presentation, remains to be elucidated. However, this differential effect of irradiation on DC function might partly explain why low doses have anti-inflammatory effects, while higher doses induce mainly proinflammatory and immune-stimulatory mechanisms [48].

In conclusion, we showed that in vivo irradiation of DCs significantly affected their functional integrity. A very important finding of our work was that low and high doses acted via different mechanisms on DCs. High-dose irradiation enhanced DC-induced immune activation by improving DC–Teff interactions by upregulating those phenotypical markers on DCs, which are responsible for more efficient T cell stimulation and by increasing the production of cytokines involved in stress response, inflammation, and immune activation. On the other hand, low-dose irradiation augmented DC-antigen uptake but altered DC-antigen presentation, which indicates a shift towards a less mature DC phenotype with reduced immune-stimulating capacity. Our data also support the model of the nonlinear dose–response relationship of the molecular events characteristic for low-dose irradiation-induced immunological alterations [63]. We think that these results help to better understand the influence of ionizing radiation on DC function, the dose dependency of the effects, and ultimately help in optimizing the combination of radiotherapy and immunotherapy. 

## 4. Materials and Methods

### 4.1. Animal Model and Irradiation Procedure

C57Bl/6 mice aged 8–13 weeks were used in the experiments. Mice were kept and investigated in accordance with the guidelines and all applicable sections of the Hungarian and European regulations and directives. All animal studies were approved and permission was issued by the Budapest and Pest County Administration Office Food Chain Safety and Animal Health Board (permit number: PE/EA/392-7/2017, 8/5/2017). Mice were total-body irradiated with 0 (control), 0.1, 0.25, and 2 Gy X-rays using THX-250 therapeutic X-ray source (Medicor, Budapest, Hungary). The energy of the X-ray source was 200 keV. Mice were sacrificed and spleens were removed either 1 or 3 days after irradiation. For DC isolation, spleens of 2 mice were pooled.

### 4.2. Isolation of Splenocytes and Dendritic Cells

Spleens were mechanically disaggregated and cell suspensions were collected and pelleted in RPMI-5 culture medium consisting of RPMI-1640 culture medium (Lonza, Basel, Switzerland) supplemented with 5% foetal bovine serum (Thermo Fischer, Waltham, MA, USA). Erythrocytes were eliminated by incubation with a lysis buffer containing 1.66% ammonium chloride. Cells were washed with RPMI-5 and passed through a 30-μm cell strainer to obtain single-cell suspension.

To release as many DCs cells as possible, spleens were digested with 400 U collagenase D (Roche, Basel, Switzerland) in Hanks’ Balanced Salt Solution (HBSS) (Sigma-Aldrich, St. Louis, USA) after mechanical disaggregation. Splenocytes were labeled with CD11c Microbeads (Miltenyi Biotec, Bergisch Gladbach, Germany) and purified by magnetic cell sorting according to the manufacturer’s instructions. Purity of isolated DCs is shown in Supplementary Figure S5. Purified DCs were resuspended either in RPMI-10 culture medium (RPMI-1640 culture medium supplemented with 10% heat-inactivated FBS) for antigen uptake and antigen presentation assays, or in an RLT lysis buffer (Qiagen, Hilden, Germany) containing 1% β mercapto-ethanol for RNA isolation.

### 4.3. Immunophenotyping of DCs

The phenotypical analysis of splenic DCs was performed using the following antimouse antibodies: CD11c-PE, CD11c-APC, and I-Ab-FITC (Biolegend, San Diego, CA, USA) for the identification of the splenic DC population; CD40-PECy5, CD80-PECy5- CD86-PECy5, B7-H1-APC, and DEC205-AlexaFluor^®^647 (Biolegend) for detecting DC-specific cell surface markers; and IL-1α-PE and IL-1β-PE (eBioscience, San Diego, CA, USA) for evaluating intracellular cytokine production by DCs. Single-cell suspensions of splenocytes were incubated with the fluorescently labeled antibodies against cell-surface markers in a staining buffer containing 1% BSA at 4 °C for 20 min. For intracellular cytokine measurements, cells were fixed and permeabilized with the FOXP3 Fix/Perm Buffer Set (BioLegend) and incubated with the corresponding anticytokine antibodies at room temperature for 30 min. All measurements were done with a CytoFLEX flow cytometer (Beckman Coulter, Brea, CA, USA) and analyzed by the CytExpert software (Beckman Coulter).

### 4.4. DC Antigen Uptake and Presentation

Antigen uptake was tested on splenic DCs isolated by magnetic separation as described above. Cells were incubated at 37 °C in the presence of an FITC-labeled SIINFEKL peptide, which is an epitope of the OVA protein (Sigma-Aldrich, St. Louis, MI, USA). To exclude unspecific uptake, the same cells incubated with the FITC-labeled peptide at 4 °C were used as background. To measure antigen presentation, splenic DCs were isolated as described above, incubated in RPMI-10 in the presence of 30 μM final concentration of unlabeled SIINFEKL peptide at 37 °C or at 4 °C for 2 h. The peptide bound to MHCI at 4 °C was considered nonspecific and was deducted. Cells were labeled with H2b-SIINFEKL-FITC antibody able to detect MHCI-bound SIINFEKL peptide.

Peptide uptake and presentation by splenic DCs was measured by a CytoFLEX flow cytometer (Beckman Coulter) and analyzed by the CytExpert software (Beckman Coulter).

### 4.5. Measuring Cytokine Gene Expression of DCs by qRT-PCR

Splenic DCs were separated by magnetic cell sorting as described above and RNA was extracted using the RNeasy Kit (Qiagen) kit according to the manufacturer’s instructions. A total of 0.5 μg RNA was subjected to cDNA synthesis using a RevertAid First Strand cDNA Synthesis Kit (Thermo Fisher Scientific, Waltham, MA, USA), according to the manufacturer’s protocol. The cDNAs were used for qRT-PCR. The qRT-PCR reaction mix was as follows: 0.25 μL cDNA, 0.45 μL forward primer (12.5 pM), 0.45 μL reverse primer (12.5 pM), 7.5 μL Maxima SYBR Green/ROX qPCR Master Mix (2X) (Thermo Fisher Scientific), and 7.3 μL water. Reactions were performed in a Rotor-Gene Q Real Time PCR Cycler (Qiagen, Hilden, Germany). The cycling profile of the reaction mix was as follows: initial denaturation at 95 °C for 15 min, 40 cycles of denaturation (95 °C, 15 s), annealing (60 °C, 30 s), and primer extension (72 °C, 30 s). The final extension was at 72 °C for 10 min. After melting-curve analysis (72–95 °C), a final reannealing was performed at 72 °C for 10 min. RT-PCR reactions were made in triplicates and five independent biological samples were analyzed. Expression patterns were normalized relative to the expression of mouse 18S RNA using the comparative Ct method in the Rotor-Gene version 2.3 software (Qiagen, Hilden, Germany). The sequences of primers used in the RT-PCR reactions are shown in Table 2. Primers were designed with Primer3 software (available online: http://frodo.wi.mit.edu/).

### 4.6. In Vivo Cytokine Assay

Mice were either sham-irradiated or irradiated with 2 Gy, and, 18 h later, 250 μg Brefeldin A (Sigma) and 100 ng LPS (Sigma) were administered intraperitoneally (i.p.). One day after irradiation, mice were sacrificed, spleens were removed, and splenocytes were labeled with fluorescent antibodies for DC markers (CD11c and MHCII) and intracellular cytokines (IL-1α and IL-1β) were measured by flow cytometry as described above.

### 4.7. T Cell-Proliferation Assay

CD4+CD25+ Tregs were purified by magnetic cell sorting using the Miltenyi Biotec’s CD4+CD25+ Regulatory T Cell Isolation Kit (Miltenyi Biotec GmbH, Bergisch Gladbach, Germany) according to the manufacturer’s instructions. Briefly, this kit isolates Tregs through 2 successive magnetic-isolation steps. First, non-CD4+ cells within the splenocyte single-cell suspensions were eliminated through indirect magnetic labelling with a Biotin-Antibody cocktail and Antibiotin microbeads. The recovered cells, highly enriched in CD4+ cells, were stained with a CD25+ PE antibody and magnetically labeled with Anti-PE microbeads. This second magnetic-isolation step allowed the recovery of the CD4+CD25+ Tregs retained on the separation columns, while the flow-through consisted of CD4+CD25− Teffs. The purity of CD4+CD25+ cells was on average 90% or above. In order to check that the isolated CD4+CD25+ cells were indeed Tregs, intracellular Foxp3 staining was performed, indicating that more than 85% of the CD4+CD25+ cells were also positive for Foxp3 (Supplementary Figure S6). Foxp3 staining of CD4 cells was performed as described previously [22]. CD4+CD25− effector T cells (Teffs) used in the experiments were isolated using the MagCellect Mouse CD4+CD25+ Regulatory T Cell Isolation Kit (R and D Systems, Minneapolis, MN, USA) according to the manufacturer’s instructions. Basically, the magnetic-isolation steps were similar to the Miltenyi Biotec’s CD4+CD25+ Regulatory T Cell Isolation kit, but the purity of CD4+CD25− cells was superior (above 95%). Either Tregs or Teffs (1 × 10^5^ cells/well, each) were cultured with or without DCs isolated from sham-irradiated or irradiated mice at a T cell:DC ratio of 1:1 in 96-well U-bottom plates for 5 days. T cell proliferation was measured by ^3^H-thymidine incorporation during the last 16–18 h of culture following the addition of 1 μCi methyl-^3^H-thymidine (MP Biomedicals Inc., Irvine, CA, USA). Cells were harvested, washed, and lysed in a Soluene-350 (Perkin Elmer, Waltham, MA, USA) lysis buffer (4 parts soluene, 1 part water), added in an Ultima Gold (Perkin Elmer) scintillation cocktail. The uptake of radioactivity was measured using the Packard Tri-Carb 2900 TR liquid-scintillation counter (Perkin Elmer) and expressed in counts per minute (cpm).

### 4.8. Statistical Analysis

Student’s *t*-test was applied to determine statistical significance using GraphPad Prism version 6.00 for Windows (GraphPad Software, La Jolla, CA, USA, available online: www.graphpad.com). Data are presented as mean ± standard deviation (SD), and were considered statistically significant if *p* value was lower than 0.05. 

## Figures and Tables

**Table 1:** Relative changes in the phenotype of splenic DCs irradiated with 2 Gy and/or treated with lipopolysaccharide (LPS) compared to control mice 3 days after irradiation. Data represent the average of four independent experiments (2–4 animals for each experiment) for each dose point. Significance compared to 0 Gy DC was tested by Student’s *t*-test (* *p* < 0.05, ** *p* < 0.01).

Treatment	CD40+	CD80+	CD86+	B7-H1+	DEC205+
0 Gy DC	1.00 (±0.37)	1.00 (±0.05)	1.00 (±0.42)	1.00 (±0.12)	1.00 (±0.24)
0 Gy DC + LPS	3.45 (±2.88)	1.54 (±0.39) *	2.35 (±1.90)	1.25 (±0.13)	1.09 (±0.37)
2 Gy DC	2.82 (±0.87) **	1.08 (±0.62)	1.50 (±0.80)	1.02 (±0.19)	1.38 (±0.26)
2 Gy DC + LPS	2.95 (±1.38) *	1.56 (±0.46)	2.18 (±1.89)	1.29 (±0.18)	1.27 (±0.39)

**Table 2:** Sequence of primers used for qRT-PCR assays.

Name (Product Length)	Sequence
18S RNA (199 bp)	forward: 5′-CGCGGTTCTATTTTGTTGGT-3′
	reverse: 5′-AGTCGGCATCGTTTATGGTC-3′
IL-1α (161 bp)	forward: 5′-CCCGTCCTTAAAGCTGTCTG -3′
	reverse: 5′-AATTGGAATCCAGGGGAAAC -3′
IL-1β (230 bp)	forward: 5′-GCCCATCCTCTGTGACTCAT -3′
	reverse: 5′-AGGCCACAGGTATTTTGTCG -3′
IL-6 (134 bp)	forward: 5′-ATCCAGTTGCCTTCTTGGGACTGA-3′
	reverse: 5′-TAAGCCTCCGACTTGTGAAGTGGT-3′
IL-10 (119 bp)	forward: 5′-GGGTTGCCAAGCCTTATCGGAAAT-3′
	reverse: 5′-TCTTCAGCTTCTCACCCAGGGAAT-3′
IL-12 (210 bp)	forward: 5′-GAAGTCCAATGCAAAGGCGGGAAT-3
	reverse: 5′-AAAG-CCAACCAAGCAGAAGACAGC-3′
TNFα (143 bp)	forward: 5′-CCAACGGCATGGATCTCAAAGACA-3′
	reverse: 5′-TGAGATAGCAAATCGGCTGACGGT-3′

## Supplementary Materials

Materials can be found at http://www.mdpi.com/1422-0067/19/8/2391/s1.
