# Supplementary material for: In Vivo Irradiation of Mice Induces Activation of Dendritic Cells
Source: Int J Mol Sci. 2018 Aug 14;19(8):2391. doi: 10.3390/ijms19082391 (PMC6121955; doi:10.3390/ijms19082391)
Supplement: Supplementary file 1 [file ijms-19-02391-s001.pdf]

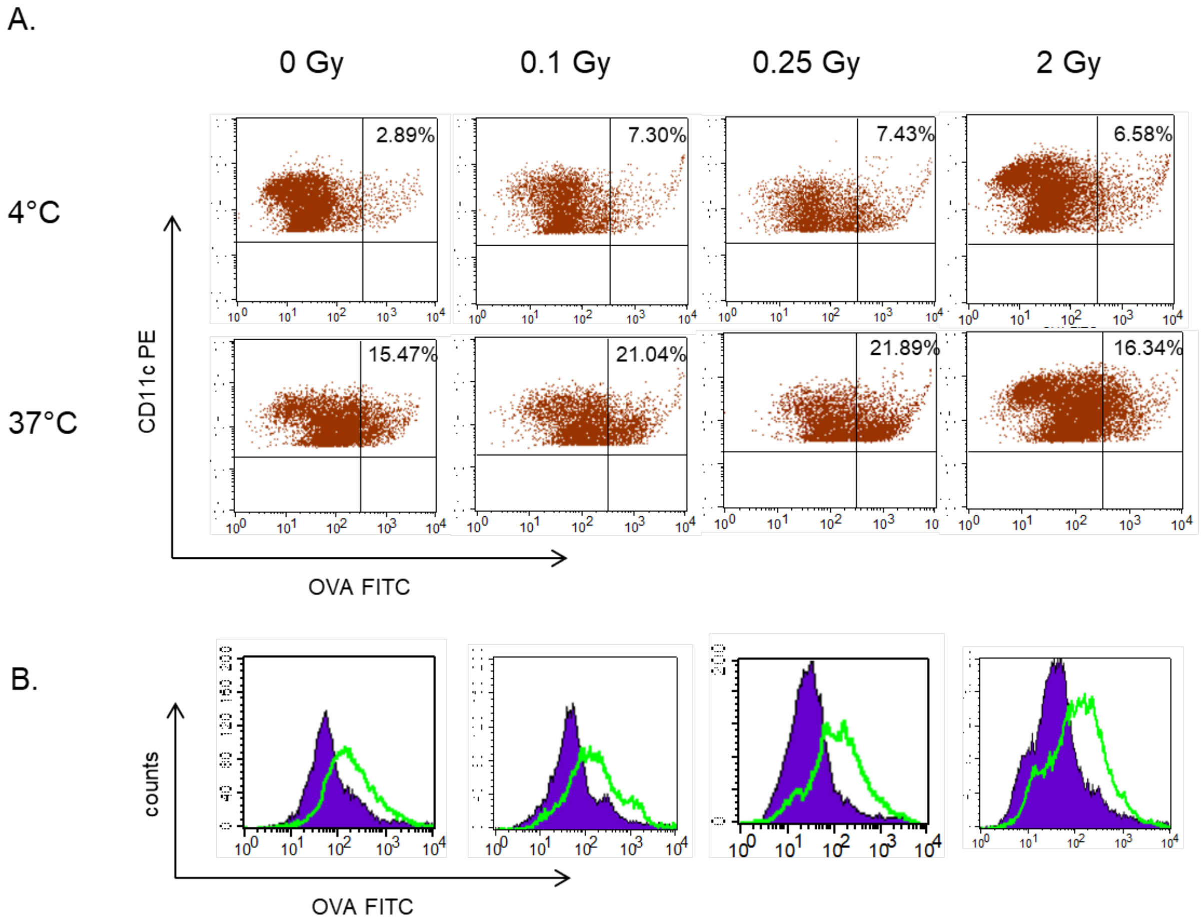

Supplementary figure 1. Ovalbumine (OVA) peptide uptake by CD11c+MHCII+ DCs. **A.** Isolated dendritic cells (DCs) were incubated with FITC-labeled OVA peptide at 4°C and at 37°C simultaneously, as described in Materials and Methods. The percentage of OVA peptide uptake by CD11c+MHCII+ DCs was calculated by subtracting values of unspecific uptake observed at 4°C. **B.** Histogram plots of DC fluorescence after FITC-labeled OVA peptide uptake expressed as mean fluorescent intensities (MFI) in control samples (left histogram) and samples irradiated with 2 Gy (right histogram). Differences between samples incubated in 4°C (purple areas) and 37°C (green lines) are shown.

A.

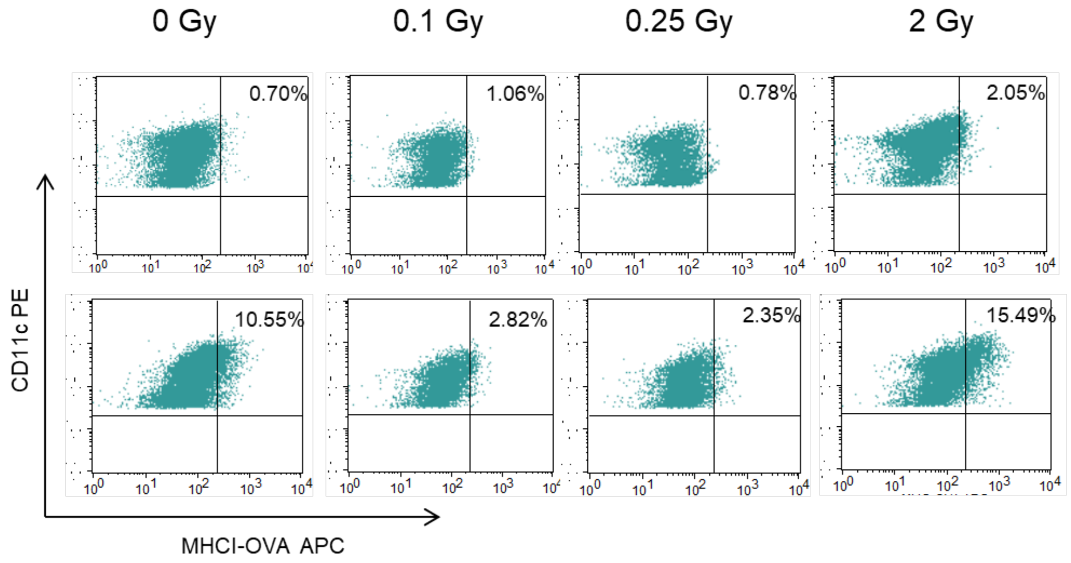

B.

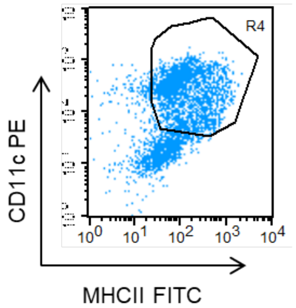

Supplementary figure 2. OVA peptide presentation on major histocompatibility complex (MHCI) molecules by CD11c+MHCII+ DCs. Isolated DCs were incubated in the presence of unlabeled SIINFEKL peptide. Cells were labeled with H2b-SIINFEKL-FITC antibody able to detect MHCI-bound SIINFEKL peptide. **A.** The percentage of OVA peptide presentation by DCs was calculated by subtracting values of unspecific binding observed at 4°C. **B.** Gating strategy shown for CD11c+MHCII+ cells.

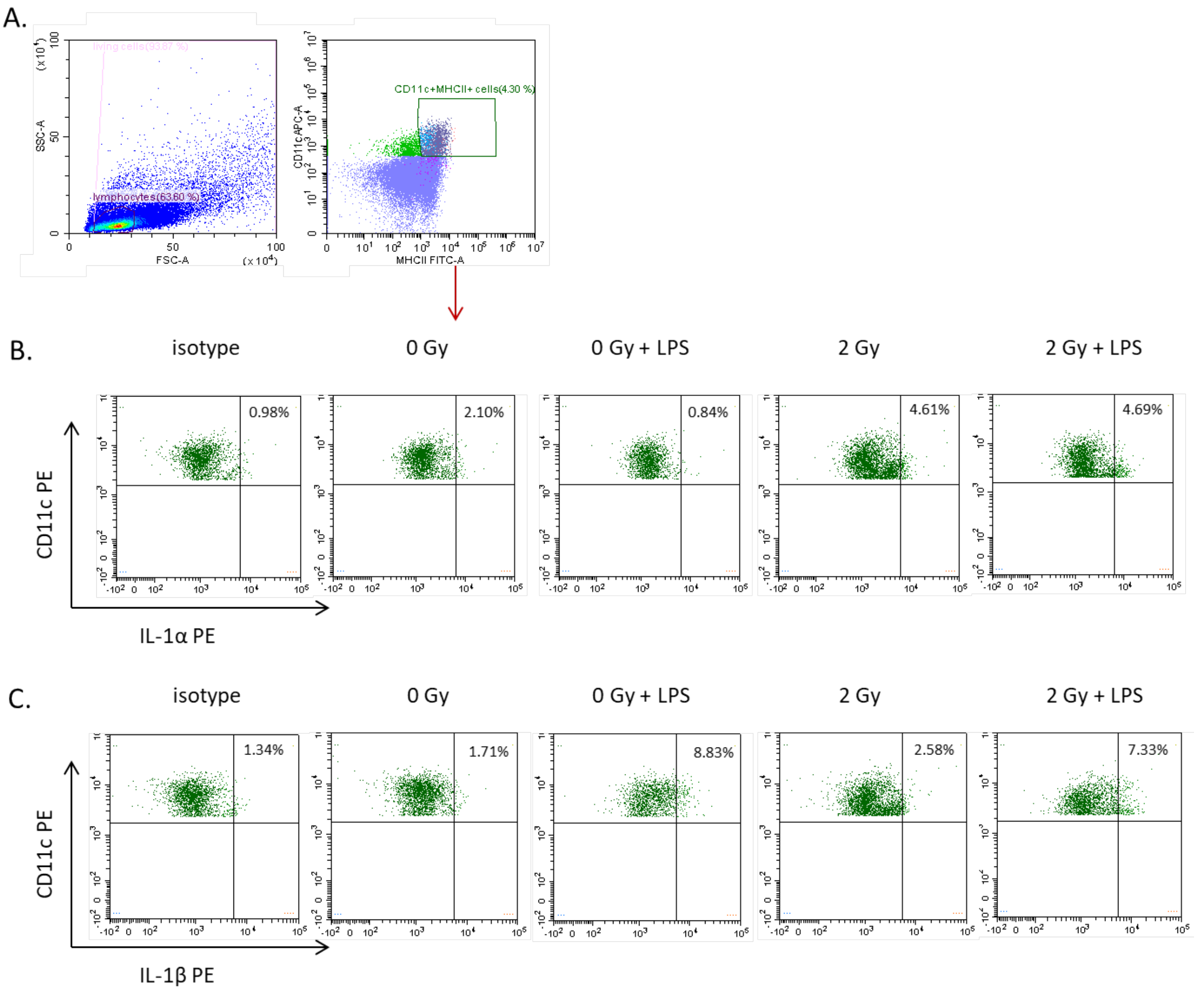

Supplementary figure 3. A. Gating strategy of CD11c+MHCII+ cells within splenocytes with lymphocyte population being excluded. Dot plots of (**B.**) IL1α and (**C.**) IL1β levels. The plot shows the percentage of cytokine expression of control (0 Gy) and irradiated (2 Gy) and/or lipopolysaccharide (LPS)-stimulated CD11c+MHCII+ DCs. Mice were total-body irradiated, treated with intraperitoneal (i.p.) LPS injection 18 hours later, followed by i.p. Brefeldin A injection. Cytokine production was determined 6 hours after LPS treatment in the CD11c+MHCII+ splenic DCs by intracellular cytokine labelling as described in Materials and Methods. Data represent one typical experiment.

Supplementary table 1. Treg and Teff cells' proliferation measured in counts per minute (cpm). Either isolated Tregs or Teffs were cultured with DCs isolated from sham-irradiated or irradiated mice as described in Materials and Methods. T cell proliferation was measured by 3H-thymidine incorporation using a liquid-scintillation counter and expressed in cpm.

**Counts per minute (CPM)**		0 Gy DC		2 Gy DC	
		Teff	Treg	Teff	Treg
experiment 1	sample 1	985	1738	2260	846
	sample 2	1458	3578	2925	2505
	sample 3	1921	3578		2855
experiment 2	sample 1	4382	5060	4593	3706
	sample 2	5481	6873	4549	4256
	sample 3	6518	6431	7122	3830
	sample 4	5921	6023	5179	2324
experiment 3	sample 1	1880	2253	1791	2518
	sample 2	1651	2413	2676	2421
	sample 3	2432	3305	2459	2154
	sample 4	1888	2652	1693	1573
Average		**3037.91**	**3991.27**	**3524.7**	**2635.27**
Standard deviation		2024.85	1808.94	1765.21	999.20

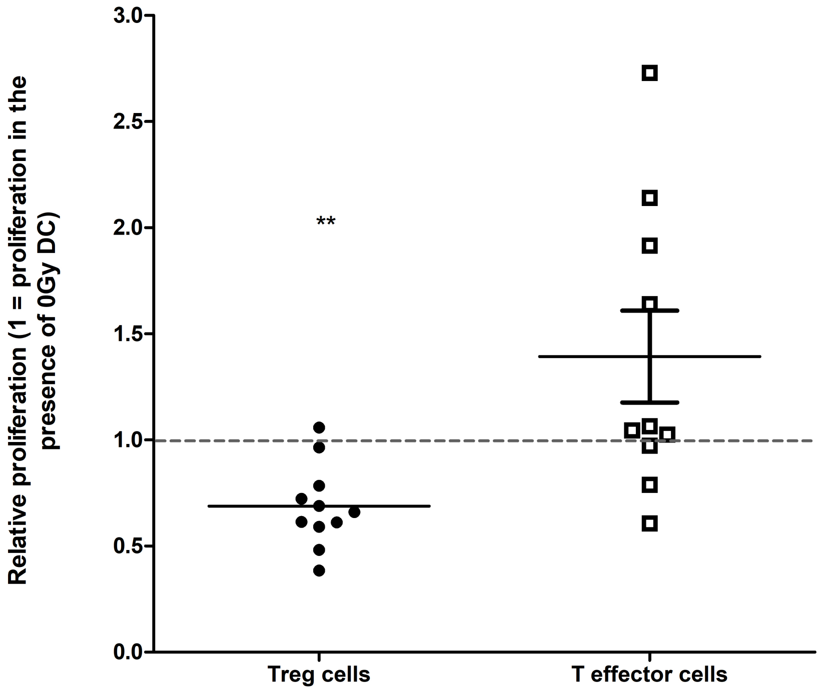

Supplementary figure 4. Relative proliferation of Tregs and Teffs after coculture with 2 Gy DCs. Isolated Treg and Teff cells were incubated with 0 Gy and 2 Gy DCs. Proliferation was measured 5 days later by ^{3}H-thymidine incorporation as described in Materials and Methods. Data represent the average of three independent experiments, with bars indicating mean ± SD. Significance was tested by Student's t-test with **p<0.001.

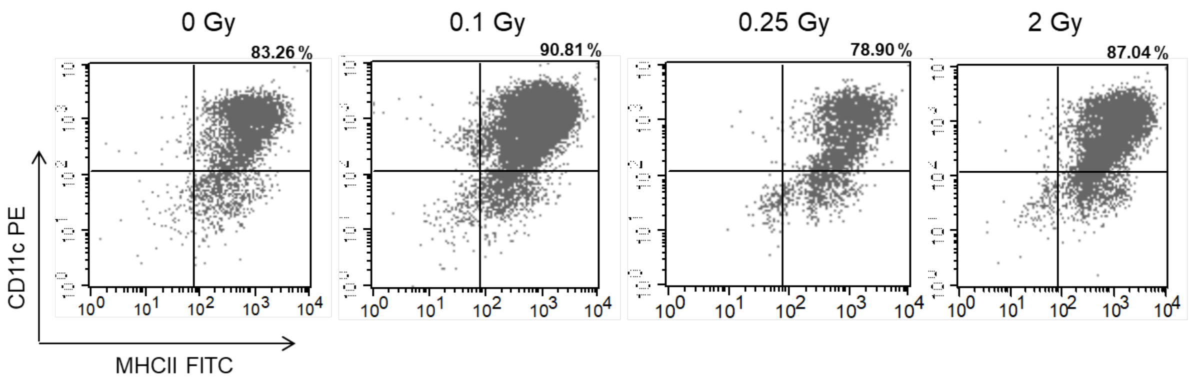

Supplementary figure 5. Dot plots showing the purity of DC-cell population. CD11c+ cells were purified by magnetic cell sorting using the Miltenyi Biotec's CD11c+ Dendritic Cell Isolation Kit, as described in Materials and Methods. Isolated CD11c+ cells were labelled with CD11c-PE and MHCII-FITC antibody. Data are from one representative experiment.

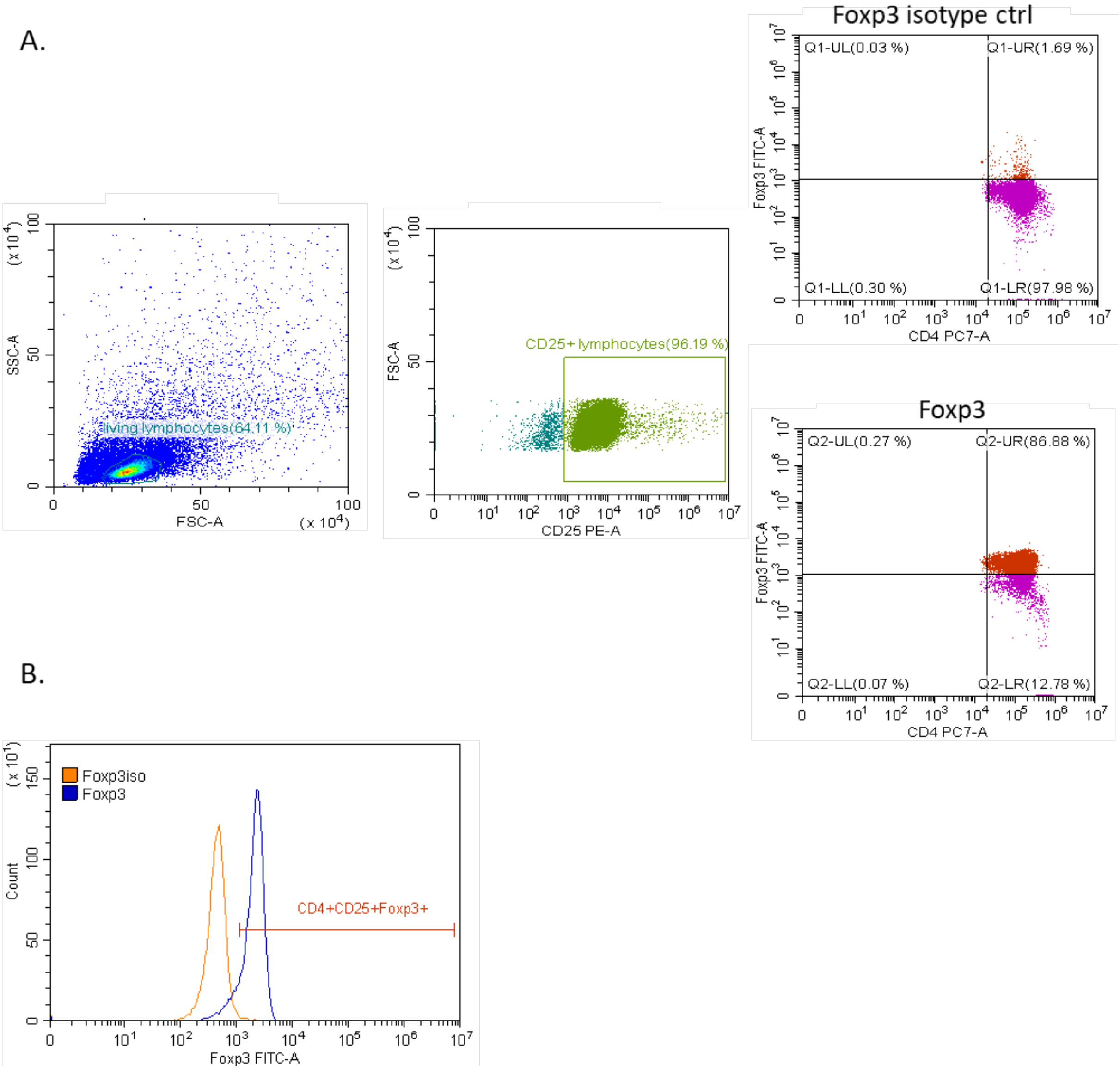

Supplementary figure 6. (**A.**) Dot plot and (**B.**) histogram data showing the purity of isolated Treg cells. CD4+CD25+ Tregs were purified by magnetic cell sorting using the Miltenyi Biotec's CD4+CD25+ Regulatory T Cell Isolation Kit, as described in Materials and Methods. Isolated CD4+CD25+ cells were fixed, permeabilized, and labeled intracellularly with a Foxp3-FITC antibody. Percentage of Foxp3+ cells within CD4+CD25+ cell population represents purity of isolated Treg cells. Data are from one typical experiment.
